# Digital PCR detection of plasmid DNA administered to the skeletal muscle of a microminipig: a model case study for gene doping detection

**DOI:** 10.1186/s13104-018-3815-6

**Published:** 2018-10-10

**Authors:** Teruaki Tozaki, Shiori Gamo, Masaki Takasu, Mio Kikuchi, Hironaga Kakoi, Kei-ichi Hirota, Kanichi Kusano, Shun-ichi Nagata

**Affiliations:** 1 Genetic Analysis Department, Laboratory of Racing Chemistry, 1731-2 Tsurutamachi, Utsunomiya, Tochigi 320-0851 Japan; 2 Department of Veterinary Medicine, Faculty of Applied Biological Sciences, Gifu University, 1-1 Yanagido, Gifu, Gifu 501-1193 Japan; 3 Racehorse Hospital Ritto Training Center, Japan Racing Association, 1028 Misono, Ritto, Shiga 520-3085 Japan

**Keywords:** Gene doping, Horseracing, Plasmid, Thoroughbred

## Abstract

**Objective:**

Doping control is an important and indispensable aspect of fair horse racing; genetic doping has been recently included to this. In this study, we aimed to develop a detection method of gene doping. A plasmid cloned with human *erythropoietin* gene (p.hEPO, 250 μg/head) was intramuscularly injected into a microminipig. Subsequently, p.hEPO was extracted from 1 mL of plasma and detected by droplet digital polymerase chain reaction.

**Results:**

The results confirmed that the maximum amount of plasmid was detected at 15 min after administration and the majority of the plasmid was degraded in the bloodstream within 1–2 days after administration. In contrast, low amounts of p.hEPO were detected at 2–3 weeks after administration. These results suggest that the proposed method to detect gene doping can help obtain information for experiments using horses.

## Introduction

During the early 1700s, horseracing was introduced in Britain with the development of thoroughbred horses by mating among Arabian stallions and British native mares [1]. As the horse racing industry has three main aspects, viz., breeding, racing, and wagering, fair horseracing management is extremely important. This can be accomplished through doping control.

The International Federation of Horseracing Authorities (IFHA) recently defined genetic therapy for racehorses to control gene doping [2]. Genetic therapy is defined as “the administration of oligomers or polymers of nucleic acid and nucleic acid analogues”. In this context, polymers of nucleic acids and their analogues are considered as transgenes [3, 4], which have been linked to athletic performance in horses [5]. Therefore, the development of detection methods for these polymers is important to control gene doping. Several approaches, such as protein or transgene detection methods, have been considered for the detection of gene doping [6, 7].

Studies on the clearance of plasmids often use small experimental animals, such as mice [8, 9]; however, there are only a few studies on medium- and/or large-sized animals. As the body size is significantly different between mouse and horse, it is not preferable to directly use the information obtained from mouse for horse, i.e., the amount of plasmid vector administered should be based on body weight conversion between them. In this study, the microminipig, which is a medium-sized animal, was used to investigate the clearance of plasmid DNA from blood, in order to apply the information obtained to horses. In this study, we aimed to develop a detection method of gene doping.

## Main text

### Ethical considerations

This study was approved by the Committee for Animal Research and Welfare of Gifu University (No. 17122) and was conducted at the facility in Gifu University. Minimum number of animals was used in this model case study because of animal ethics and welfare. Furthermore, plasmid administration and blood sampling were performed after anesthetizing the animals to ensure that they are not stressed.

### Materials and methods

The human *erythropoietin* (EPO) gene cloned into plasmid vector (p.hEPO) was procured (OriGene, Rockville, MD, USA), and then a large amount of p.hEPO was purified by transforming into *Escherichia coli* competent cell, JM109 (Takara Bio Inc., Shiga, Japan). The concentration (ng/μL) of purified p.hEPO was measured using the Qubit dsDNA HS Assay Kit (Thermo Fisher Scientific, Waltham, MA, USA) and the copy number was measured by the droplet digital polymerase chain reaction (ddPCR; Bio-Rad, Hercules, CA, USA).

To detect p.hEPO by the ddPCR, the following TaqMan-MGB probe and primers were synthesized; probe (P3/4): CGACCTCCATCCTCTTC, forward-primer (F3): TCCCAGACACCAAAGTTAATTTC, reverse-primer (R4): CCTGCCAGACTTCTACGG (Thermo Fisher Scientific). These were designed according to Baoutina et al. [7] to detect human *EPO* gene in the gene doping detection experiment.

For ddPCR, a method recommended by the manufacturer was used: 8.8 μL of sample solution, 11 μL of ddPCR Supermix for Prove (no dUTP), 0.2 μL of 100 μM F3-primer, 0.2 μL of 100 μM R4-primer, 0.6 μL of 10 μM P3/4-probe in the total volume of 22 μL. After creating a droplet with an Automated Droplet Generator (Bio-Rad), the PCR was carried out under the following conditions: enzyme activation reaction at 95 °C for 10 min, 40 cycles of denaturation reaction at 94 °C for 30 s and annealing/extension at 60 °C for 1 min. After enzyme deactivation for 10 min at 98 °C, the PCR products were stored at 12 °C. Subsequently, the samples were measured using the QX200 Droplet Reader (Bio-Rad). Each ddPCR was performed using negative template controls (NTCs: Mili-Q water or extracts from blank plasma) and positive template controls (PTCs: serially diluted p.hEPO).

One microminipig (male, 8-month old; 9.2 kg, FujiMicra, Shizuoka, Japan) was intramuscularly injected 250 μg of p.hEPO, and then blood was sampled. Blood sampling was performed at 15 min, 3 h, 6 h, 12 h, 1 day, 2 days, 3 days, 4 days, 5 days, 7 days, 2 weeks, and 3 weeks after administration. Four milliliters of blood was collected in an EDTA blood collection tube.

Immediately after sampling, the collected blood was centrifuged, and the plasma was separated and stored at − 20 °C. Then, p.hEPO was extracted from 1 mL of plasma with Chemagic Prepito (PerkinElmer, Waltham, MA, USA) using the Prepito Circulating NA 1 K Kit (PerkinElmer) by the magnetic bead method. The extract finally eluted is approximately 90 μL. In this study, the elution buffer provided in the kit was not used; instead we used Mili-Q. The recovery rate obtained by the ddPCR was ~ 60% by the spike/recovery assays (data not shown).

### Results and discussion

**Fig. 1:**
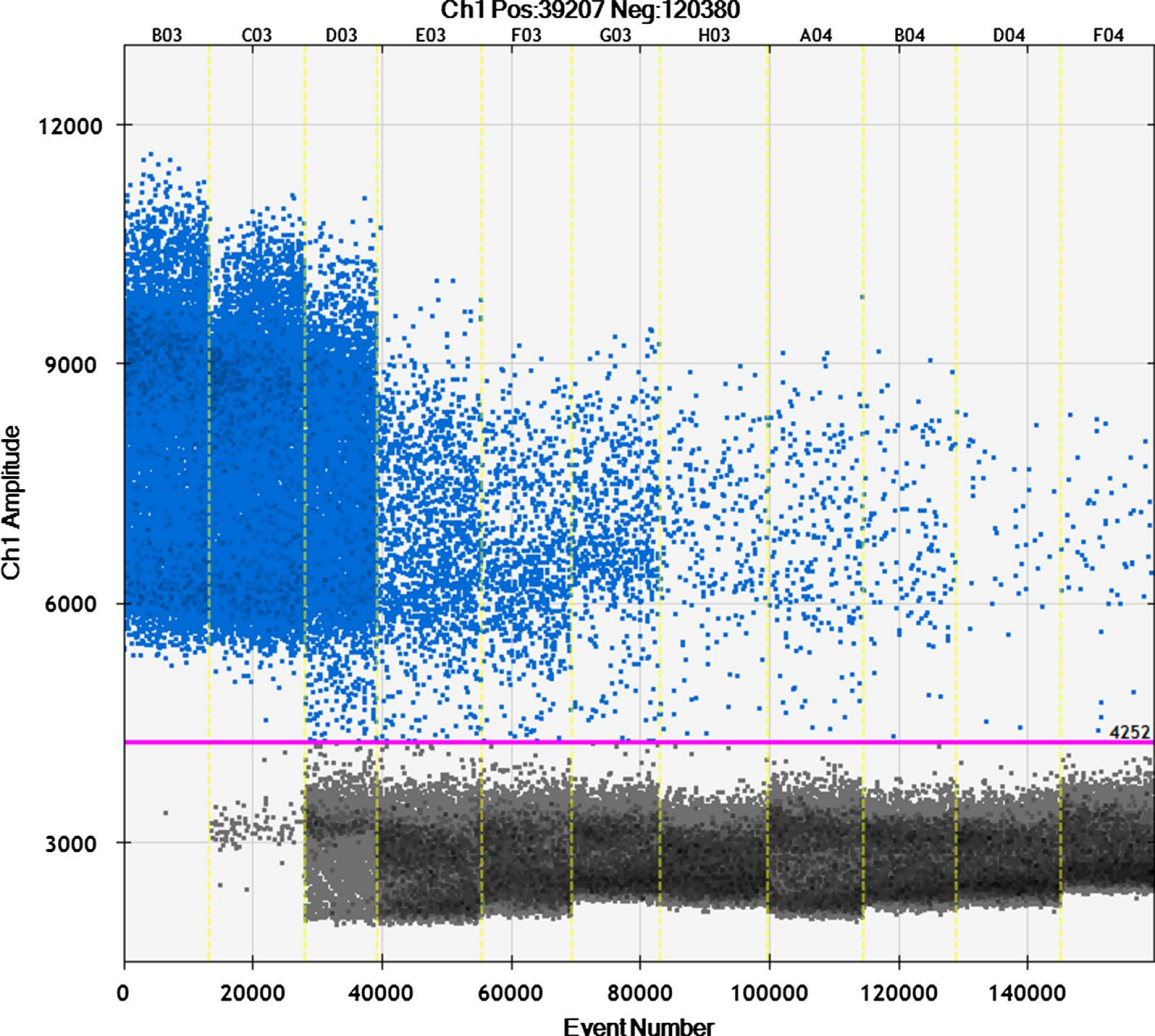
Detection of p.hEPO in the plasma by ddPCR. Horizontal axis shows each sample (15 min, 6 h, 1 day, 2 days, 3 days, 4 days, 5 days, 6 days, 7 days, 2 weeks, and 3 weeks) after the administration of p.hEPO and vertical axis shows the amplitude of ddPCR products. Threshold line for non-amplified/amplified was manually defined at the amplitude of 4252. At 15 min after administration, 2.52 × 10^6^ copies in 1 mL plasma were detected, and then a large amount of p.hEPO was degraded in the blood within 1–2 days after administration

Figure 1 and Table 1 present the detection results of the ddPCR. At 15 min after administration, 2.5 × 10^6^ copies were detected in 1 mL of plasma. Immediately after intramuscular injection, a large amount of p.hEPO circulated through the blood, and then was degraded in the blood within 2 days after the administration of p.hEPO.

**Table 1:** Copy numbers detected in 1 mL of plasma by ddPCR

Collection time	Copies in 1 mL of plasma
15 min	2.52 × 10^6^
6 h	1.34 × 10^6^
1 day	2.89 × 10^5^
2 days	2.48 × 10^4^
3 days	1.55 × 10^4^
4 days	1.32 × 10^4^
5 days	3.80 × 10^3^
6 days	4.25 × 10^3^
7 days	2.52 × 10^3^
2 weeks	698
3 weeks	1.06 × 10^3^

Subsequently, a small amount of p.hEPO could be detected for 2–3 weeks after administration. We detected over 7 × 10^2^ copies in 1 mL of plasma at 2 weeks and ~ 1000 copies in 1 mL of plasma at 3 weeks after administration. This might be due to the sustained release of p.hEPO remaining at the site of injection. It was indicated that almost all the plasmids were degraded in at least 1–2 days.

Our results suggested that plasmid DNA administrated into body could be detected a few weeks after intramuscular injection. Therefore, the detection method used in this study (using 1 mL of EDTA plasma as a target substance and detection with ddPCR after DNA extraction) could help monitor gene therapy or detect gene doping.

In this study, 250 μg of p.hEPO was administered to a microminipig (medium-sized animal) of body weight 9.2 kg. As the average body weight of a 3-year-old thoroughbred racehorse (large sized-animal) is 473.9 kg [10], 250 μg of p.hEPO should correspond to approximately 12.9 mg in horses based on the body weight conversion. Therefore, if a horse is administered approximately 13 mg of p.hEPO, the substance administered might be detected for approximately 2–3 weeks. During tendinitis treatment [11], the amount of plasmid detected might decrease compared with that observed in this study, as the amount of plasmid administered is only ~ 5 mg/horse.

### Conclusion

It was demonstrated that the administered plasmid to animals could be detected in their blood samples by ddPCR, although this study was a model case study using a microminipig, which is a medium-sized animal. Therefore, it was considered that a similar approach is useful for gene doping detection in thoroughbred racehorses. Moreover, a suitable amount of plasmid that can be administered to horses can be calculated by appropriate conversion based on the body weight of horse and microminipig.

## Limitations

The limitation of this study is a case report that used one animal, microminipig, based on animal ethics. Therefore, studying with large sample size is recommended.

## Abbreviations

ddPCR: droplet digital polymerase chain reaction
EPO: erythropoietin gene
